# Short-Term Apixaban for Documented Left Atrial Appendage Thrombus in High-Risk Atrial Fibrillation Patients Undergoing Left Atrial Appendage Occlusion

**DOI:** 10.1055/s-0040-1718585

**Published:** 2020-10-31

**Authors:** Wern Yew Ding, Gregory Y.H. Lip, Timothy Fairbairn, Sukumaran Binukrishnan, Afshin Khalatbari, Periaswamy Velavan, Dhiraj Gupta

**Affiliations:** 1 Liverpool Centre for Cardiovascular Science, University of Liverpool and Liverpool Heart and Chest Hospital, Liverpool, United Kingdom; 2 Department of Clinical Medicine, Aalborg Thrombosis Research Unit, Aalborg University, Aalborg, Denmark; 3 Department of Cardiology, Liverpool Heart and Chest Hospital, Liverpool, United Kingdom

Stroke prevention with left atrial appendage (LAA) occlusion is an important alternative treatment for high-risk atrial fibrillation (AF) patients with contraindications to oral anticoagulation (OAC). 1 2 The presence of LAA thrombus detected on preprocedural imaging usually precludes implantation of the endocardial device because of a prohibitively high periprocedural embolic risk. 3 Although successful resolution of LAA thrombus with OAC has been described, all previous reports are in patients without contraindications to OAC. 4 5 6 Furthermore, long-term data in these patients are lacking. We hypothesized that short-term OAC with apixaban could safely facilitate resolution of LAA thrombi even in a high-risk population, thereby allowing safe LAA occluder device implantation, with good outcomes over the long term.

This was a retrospective cohort study of 83 patients who underwent work-up for LAA occlusion at our center between January 2015 and December 2017. LAA thrombi were documented in 11 (13.3%) patients (mean age: 76.9 [ ±  6.9] years; 7 males) on preprocedural imaging (9 on transesophageal echocardiography and 2 on cardiac computed tomographic angiography) ( Table 1 ). All patients with LAA thrombi had permanent AF, with median CHA _2_ DS _2_ -VASc and HAS-BLED scores of 4 (interquartile range [IQR]: 3–5) and 2 (IQR: 2–3), respectively. Contraindications to lifelong OAC in this cohort were prior intracranial hemorrhage in six patients (two on warfarin, one on dabigatran, one on heparin, and two while not on anticoagulation), prior major gastrointestinal hemorrhage requiring emergency hospital admission and blood transfusion in three patients (two on warfarin and one while not on anticoagulation), severe unexplained anemia on dabigatran in one patient, and failed OAC in one patient. At the time of imaging, none of the patients were receiving OAC, and four (36.4%) patients were on a single antiplatelet agent. There were no significant differences between patients who had documented LAA thrombus compared with those without in terms of age ( *p* = 0.80), sex category ( *p* = 0.74), AF type ( *p* = 0.11), comorbidities (hypertension [ *p* = 0.50], diabetes mellitus [ *p* = 0.31], and prior stroke or transient ischemic attack [ *p* = 0.47]), and CHA _2_ DS _2_ -VASc score ( *p* = 0.69).

**Table 1:** Characteristics of patients with left atrial appendage thrombus

Patients	Characteristics
Patient 1	Age: 82 y; eGFR: 42; CHA _2_ DS _2_ -VASc: 3; HAS-BLED: 2; recurrent GI bleed on warfarin; OGD revealed gastric angiodysplasia; CKD; COPD; angina
Patient 2	Age: 76 y; eGFR: 74; CHA _2_ DS _2_ -VASc: 5; HAS-BLED: 2; recurrent GI bleed on warfarin; no source identified on OGD and colonoscopy
Patient 3	Age: 75 y; eGFR: 90; CHA _2_ DS _2_ -VASc: 4; HAS-BLED: 3; massive GI bleed while off anticoagulation; OGD identified variceal source, which was treated with banding; nonalcoholic liver cirrhosis; old inferior MI; mild LV dysfunction; hypothyroidism
Patient 4	Age: 78 y; eGFR: 53; CHA _2_ DS _2_ -VASc: 5; HAS-BLED: 2; ICH on heparin for newly diagnosed atrial fibrillation; prior ischemic stroke; CKD; HTN; hypothyroidism
Patient 5	Age: 81 y; eGFR: 64; CHA _2_ DS _2_ -VASc: 3; HAS-BLED: 3; ICH on warfarin (subtherapeutic international normalized ratio); meningioma; epilepsy; HTN; prior pneumothorax
Patient 6	Age: 80 y; eGFR: 48; CHA _2_ DS _2_ -VASc: 4; HAS-BLED: 3; ICH on dabigatran; CKD; epilepsy; uncontrolled HTN; treated breast cancer
Patient 7	Age: 68 y; eGFR: 47; CHA _2_ DS _2_ -VASc: 6; HAS-BLED: 2; ICH while off anticoagulation; cerebral amyloid angiopathy; prior stroke; old MI; severe LV dysfunction; CKD; HTN
Patient 8	Age: 88 y; eGFR: 67; CHA _2_ DS _2_ -VASc: 3; HAS-BLED: 2; large ICH on warfarin; HTN
Patient 9	Age: 67 y; eGFR: 75; CHA _2_ DS _2_ -VASc: 5; HAS-BLED: 3; ICH while off anticoagulation; recurrent stroke and transient ischemic attack; prior pulmonary embolism and deep venous thrombosis; seizures; left ventricular hypertrophy
Patient 10	Age: 83 y; eGFR: 44; CHA _2_ DS _2_ -VASc: 4; HAS-BLED: 2; severe anemia of unknown origin while on dabigatran; CKD; left bundle branch block; HTN
Patient 11	Age: 68 y; eGFR: 67; CHA _2_ DS _2_ -VASc: 5; HAS-BLED: 3; ischemic stroke on apixaban; cerebral amyloid angiopathy; prior embolic stroke; polycythemia; COPD; alcohol excess; HTN

Abbreviations: CKD, chronic kidney disease; COPD, chronic obstructive pulmonary disease; eGFR, estimated glomerular filtration rate; GI, gastrointestinal; HTN, hypertension; ICH, intracranial hemorrhage; LV, left ventricular; MI, myocardial infarction; OGD, esophagogastroduodenoscopy.

The potential risks of surgical thrombectomy and ligation were felt to outweigh the benefits. Hence, following detection of LAA thrombi, off-label dose-adjusted apixaban (2.5 mg twice daily for three patients and 5 mg twice daily for eight patients) was prescribed for each patient. The choice of anticoagulation was based on evidence of potential favorable characteristics of apixaban compared with other OAC in terms of efficacy and safety profile. 7 Repeat imaging in the form of transesophageal echocardiography, or cardiac computed tomographic angiography was scheduled at 6- to 8-week intervals. Complete resolution of LAA thrombus was observed in 10 (90.9%) patients after apixaban treatment for a median of 94 days (IQR: 44–126 days). During treatment with apixaban, one patient (who received a dose of 5 mg twice daily) had a severe gastrointestinal bleed requiring blood transfusion, and one patient suffered an ischemic stroke with subsequent full recovery. There was no significant difference in the risk of bleeding according to apixaban dosage ( *p* = 0.73). One patient (patient 8, Table 1 ) had persistent LAA thrombus on repeated imaging, and a patient-centered decision was ultimately taken for continued apixaban therapy despite prior major intracranial bleed while on warfarin. In this patient, no bleeding complications were observed over a follow-up period of 25 weeks.

The 10 patients with thrombus resolution underwent successful LAA occlusion with no periprocedural complications. Apixaban was replaced by dual-antiplatelet therapy with aspirin and clopidogrel for 6 weeks postprocedure followed by single (or no) antiplatelet therapy. The decision to discontinue long-term antiplatelet therapy in three patients was based on an individualized approach after accounting for their high bleeding risk. All patients underwent cardiac imaging with transesophageal echocardiography or cardiac computed tomographic angiography at 6 to 8 weeks postprocedure; no device-related thrombus was observed in any patient. Across a median follow-up of 2.5 years (IQR: 0.6–3.3 years), one patient suffered a transient ischemic attack and another had an episode of severe epistaxis despite not being on antiplatelet or OAC therapy at the time. During this same period, four (40%) patients died.

Given the low number of patients, our study was underpowered and should therefore be interpreted with caution. Furthermore, the incidence of LAA thrombus in our study may not reflect those of the general AF population as many of our high-risk patients were not on anticoagulation therapy at baseline. It is also worth noting that we adopted a strategy of minimizing the exposure to OAC in this high-risk cohort by performing LAA occlusion without delay in patients with a confirmed resolution of LAA thrombi.

Our experience suggests that short-term treatment with apixaban may be effective and safe for aiding the resolution of documented LAA thrombus in high-risk patients who are ineligible for lifelong OAC therapy. This strategy allows the LAA occlusion procedure to be undertaken safely, thereby potentially reducing the long-term risk of thromboembolism.

## References

[1] Freeman J V Varosy P Price M J The NCDR Left Atrial Appendage Occlusion Registry J Am Coll Cardiol 2020 75 13 1503 1518 32238316 10.1016/j.jacc.2019.12.040 PMC7205034

[2] Ding W Y Mandrola J Gupta D Left atrial appendage occlusion: past, present and future Thromb Haemost 2020(e-pub ahead of print). 10.1055/s-0040-1714654 32717758

[3] De Backer O Arnous S Ihlemann N Percutaneous left atrial appendage occlusion for stroke prevention in atrial fibrillation: an update Open Hear 2014 1 01 e000020 10.1136/openhrt-2013-000020 PMC4195925 25332785

[4] Fleddermann A Eckert R Muskala P Hayes C Magalski A Main M L Efficacy of direct acting oral anticoagulant drugs in treatment of left atrial appendage thrombus in patients with atrial fibrillation Am J Cardiol 2019 123 01 57 62 30376957 10.1016/j.amjcard.2018.09.026

[5] Piotrowski R Zaborska B Pilichowska-Paszkiet E Sikora-Frąc M Baran J Kułakowski P RIVAroxaban TWICE daily for lysis of thrombus in the left atrial appendage in patients with non-valvular atrial fibrillation: the RIVA-TWICE study Arch Med Sci 2019 16 02 289 296 32190138 10.5114/aoms.2019.86616 PMC7069443

[6] X-TRA study and CLOT-AF registry investigators Lip G YH Hammerstingl C Marin F Left atrial thrombus resolution in atrial fibrillation or flutter: Results of a prospective study with rivaroxaban (X-TRA) and a retrospective observational registry providing baseline data (CLOT-AF) Am Heart J 2016 178 126 134 27502860 10.1016/j.ahj.2016.05.007

[7] Lip G YH Mitchell S A Liu X Relative efficacy and safety of non-Vitamin K oral anticoagulants for non-valvular atrial fibrillation: Network meta-analysis comparing apixaban, dabigatran, rivaroxaban and edoxaban in three patient subgroups Int J Cardiol 2016 204 88 94 26655548 10.1016/j.ijcard.2015.11.084

